# Characterization, antimicrobial and cytotoxic activity of polymer blends based on chitosan and fish collagen

**DOI:** 10.1186/s13568-022-01433-7

**Published:** 2022-08-04

**Authors:** Andressa Coelho Ferreira, Maria Rosa Quaresma Bomfim, Carlos Henrique de Barros da Costa Sobrinho, Daniela Talissa Lobo Boaz, Railane Da Silva Lira, Valéria Costa Fontes, Mariana Oliveira Arruda, Patrícia Maria Wiziack Zago, Carlos Alberto Alves Dias Filho, Carlos José Moraes Dias, Marilene Oliveira da Rocha Borges, Rachel Melo Ribeiro, Cícero Wellington Brito Bezerra, Rosiane Silva Penha

**Affiliations:** 1 Programa de Doutorado em Biotecnologia (RENORBIO), Universidade Federal do Maranhão (UFMA), São Luís, Brazil; 2 Universidade Ceuma, São Luís, Brazil; 3 Curso de Biomedicina, Universidade Estácio de Sá, São Luís, Brazil; 4 Faculdade de Medicina, ITPAC – Santa Inês, Maranhão, Brazil; 5 Universidade Federal do Maranhão (UFMA), São Luís, Brazil; 6 Instituto Federal de Educação, Ciência e Tecnologia do Maranhão (IFMA), S/N, Residencial Val paraíso, Sapucaia, Rosario, 65143-000 Brazil

**Keywords:** Chitosan, Fish collagen, Polymer blends, Antimicrobial activity, Cytotoxicity

## Abstract

This study aims to produce, characterize, and assess the antimicrobial activity and cytotoxicity of polymer blends based on chitosan (CT) and fish collagen (COL) produced by different precipitation methods. Polymer blends were obtained in alkaline (NaOH), saline (NaCl), and alkaline/saline (NaOH/NaCl) solutions with different CT:COL concentration ratios (20:80, 50:50, and 80:20). The polymer blends were characterized by various physicochemical methods and subsequently evaluated in terms of their in vitro antimicrobial and cytotoxicity activity. In this study, the degree of chitosan deacetylation was 82%. The total hydroxyproline and collagen content in the fish matrix was 47.56 mg. g^−1^ and 394.75 mg. g^−1^, respectively. The highest yield was 44% and was obtained for a CT:COL (80:20) blend prepared by precipitation in NaOH. High concentrations of hydroxyproline and collagen in the blends were observed when NaOH precipitation was used. Microbiological analysis revealed that the strains used in this work were sensitive to the biomaterial; this sensitivity was dose-dependent and increased with increasing chitosan concentration in the products. The biocompatibility test showed that the blends did not reduce the viability of fibroblast cells after 48 h of culture. An analysis of the microbiological activity of the all-polymer blends showed a decrease in the values of minimal inhibitory concentration (MIC) and minimal bactericidal concentrations (MBC) for *S. aureus* and *P. aeruginosa*. The blends showed biocompatibility with NIH-3T3 murine fibroblast cells and demonstrated their potential for use in biomedical applications such as wound healing, implants, and scaffolds.

## Key points

- Different precipitation methods do not change the biological properties of polymer blends.
- Gram-positive bacterias and *Pseudomonas aeruginosa* were sensitive to polymer blends.
- The blends produced showed biocompatibility in NIH-3T3 murine fibroblast cells.

## Introduction

Polymer blends are physical mixtures of two or more polymers with or without chemical bonds between the componentes (Fortelný and Jůza 2019). Most natural polymers are hydrophilic to some degree; this is due to the presence of polar groups such as hydroxyl and amine groups in their structure (Mahesh et al. 2019). The advantage of this mixture is the possibility of obtaining a new material without synthesis of a new polymer or copolymer, reducing the time spent on research, and reducing the high cost associated with the chemical synthesis and the development of these products (Dean et al. 2006). Thus, the production of these materials allows for the fabrication of a variety of products with different polymer proportions in the blend, presenting many different advantages attributed to the physicochemical characteristics of each Polymer (Quiroz-Castillo et al. 2014).

Chitosan is one of the most abundant and promising polymers, a linear, semicrystalline polysaccharide composed of β- (1 → 4) -2-amino-2-deoxy-D-glucose (glycosamine) units and shorter β- (1 → 4) chitin-2-acetamide-2-deoxy-D-glucose (N-acetylglycosamine units Dash et al. 2011; Pereda et al. 2011). Chitosan is not commonly found in the environment. However, it can easily be obtained by deacetylation of chitin, a widely distributed polysaccharide in nature and the second most abundant natural polymer (Crosier and Jérôme 2013). Chitin can be found in crustacean carapace (mainly shrimp and crab), insects (scorpions, spiders, and beetles), and fungal cell walls (Laranjeira and Fávere 2009; Lima et al. 2006).

Collagen, on the other hand, is a protein found in the connective tissues of mammals such as bones, tendons, cartilage, veins, skin, teeth, and muscles (Chen et al. 2008; Horn et al. 2010). The collagen is composed of three chains of helical polypeptides intertwined in a triple helix structure that present essential biological properties. Its principal function is to contribute to the structural integrity of the extracellular matrix or to help fix cells in the matrix, promoting tissue elasticity and resistance (Tiffany et al. 2019).

Chitosan and collagen are polymers that do not coexist as blends in nature but are miscible and can interact by forming hydrogen bonds that alternate with collagen propellers. These interactions are established between the amino groups (NH3 +) of chitosan and the carboxyl groups (COO-) of collagen (Fernandes et al. 2011) Thus, their similar biological properties, such as biocompatibility, biodegradability, bioadhesiveness, and absence of toxicity, can be improved further by mixing these two compounds, which can then be used in the production of new biomaterials (Taravel and Domard 1993). In recent years, there has been a high demand for alternative sources of substances with pharmacological activity. Indeed, both materials are now well known for their interesting physicochemical and biological properties, including their pharmacological activity (Raafat and Sahl 2009; Rodrigues, 2017). Moreover, while chitosan has interesting structural and chemical characteristics for physiological pH, and is not a natural component of the human body, collagen is the main component of the human skin and bones and one of the most abundant proteins in the human body, and, in contrast to chitosan, it is easily degraded and thermosensitive. In this way, the association between both polymers can improve their characteristics and performance as biomaterials.

Therefore, the objectives of this study were to characterize chitosan and collagen blends, produced with a new synthetic route by precipitation in alkaline, saline, and alkaline/saline solutions, by physicochemical methods and to evaluate their antimicrobial activity and cytotoxicity potential.

## Materials and methods

### Reagents and collagen extraction

Chitosan powder with 75–85% deacetylation was obtained commercially from SigmaAldrich. The swim bladder of gurijuba (*Hexanematichthys parkeri*) was obtained from a local source, and the fresh materials were taken to the laboratory, where they were washed and cooled for subsequent collagen extraction. For the preparation of all solutions, Milli-Q ultrapure water was used. All other reagents employed were of analytical grade (Aldrich and Merck) and used without further purification.

The extraction of collagen from the gurijuba swim bladder was performed as follows: first, the swim bladder was cleaned, divided, weighed (20 units/g of the swim bladder) and then prepared for acid extraction. The incubation process in acetic acid of 0.5 mol.L^−1^ was performed under constant stirring for 24 h, after which the the system was centrifuged (4 °C, 30 min) to eliminate solid particles and the collagen precipitated for addition of 3 M NaCl. The precipitate was centrifuged (4 °C, 30 min), washed with distilled water and lyophilized for over 24 h to obtain the collagen powder.

### Chitosan purification and characterization of biopolymers

To purify the chitosan, demineralization and deproteinization were performed as previously described (Santos et al. 2003). First, the samples were dissolved in 0.5 mol. L^−1^ acetic acid under stirring for 18 h, then filtered, and concentrated ammonium hydroxide was added until the precipitate formed, which was washed with distilled water to neutral pH and dried with acetone at room temperature. Subsequently, the precipitate was dried in an oven at 60 °C for 24 h.

### Determination of the degree of chitosan deacetylation (% DD)

The degree of chitosan deacetylation was determined by conductivity titration (Santos et al. 2003; Janegitz et al. 2007). For accurate measurements of conductivity, the Digimed equipment, model 21-D, was used. In the experiment, 0.2000 g of chitosan was suspended in 40 mL of hydrochloric acid solution (0.05 mol. L^−1^) for 18 h to ensure the protonation of the amino groups in the sample. The sample was titrated with NaOH standard solution (0.17 mol. L^−1^) and the conductivity was measured after each NaOH addition. To calculate the average %DD, we used the following equation:

$$\% {\text{DD}}\,{ = }\frac{{16,1\,\left[ {base} \right]\,\left( {V2 - \,\,V1} \right)}}{m}$$

where:

16,1 corresponds to the molar mass of the repetitive chitosan unit;

[base] corresponds to the concentration of the sodium hydroxide solution;

V1 corresponds to the volume of sodium hydroxide consumed to neutralize excess HCL; V2 corresponds to the base volume used to neutralize chitosan acid groups; m corresponds to the chitosan mass in the titrated sample.

### Determination of collagen 4-hydroxyproline (4-Hy) content

To determine the hydroxyproline content of the swim bladders, the amount of collagen in the samples was measured by optical spectrophotometric analysis in the visible region. The procedure was performed according to an adapted literature procedure as previously described (Neuman and Logan 1950; Huszar and Biochem 1980) using a Varian Cary 50 UV–visible spectrophotometer. Quartz cuvettes were used with an optical path length of 1 cm. Analytical curves were obtained for the following 4-Hy concentrations: 0.5, 1.0, 2.0, 3.0, and 4.0 mg. L^−1^ as previously described (Fernandes et al. 2008; Gupta and Jabrail 2006).

### Viscosity measurements

To obtain the molar mass of the polymers, viscosity tests were performed on a CannonFenske Routine capillary viscometer (Herzoo, model HVB-438) coupled with an ultrathermostatic bath (SL 152 from SOLAB). The samples were dissolved in acetic acid solution (0.5 mol L^−1^) to obtain five different sample concentrations (between 2 and 6 g. L^−1^) and their viscosity was measured at 25 ± 1 °C. Five milliliters of sample were added into the capillary, and the flow time, which is the time it takes the sample to pass through the capillary, was measured with a digital stopwatch (in seconds). Each measurement was performed in triplicate and the average of each value used for further analysis. From the analysis, the relative viscosity (ηr), specific viscosity (ηsp), and reduced viscosity (ηred) of the evaluated samples were determined. The mean viscosimetric molar mass (VM) of chitosan and collagen was calculated from the intrinsic viscosity value using the Mark-Houwink empirical equation cited by Roberts and Domszy, where* k* and* a* are constants that depend on the polymersolvent-temperature system (Gupta and Jabrail 2006; Wang and Stegemann 2010).

### Preparation and determination of chitosan (CT) and collagen (COL) blend yields

Chitosan (10 mL) and collagen (10 mL) solutions were prepared in aqueous acetic acid solution (0.5 mol/L, pH = 3). The solutions were mixed at different percent mass ratios of chitosan and collagen (CT: COL = 20:80, 50:50, and 80:20) and they were stirred for 24 h. Then, precipitation was performed with NaOH, NaCl, and NaOH/NaCl at a concentration of 3 mol.L^−1^. Subsequently, the blends were maintained at − 70 °C in a So-Low freezer (model U8518). They were then lyophilized at − 60 °C for 8 h with Terroni equipment (FauvelLB1500) (Lima et al. 2006; SPSS 2011; Wayne 2011). To determine the yield obtained via this process, 30 mg of collagen and 30 mg of chitosan were initially weighed and used to prepare CT:COL blends. At the end of the biomaterial preparation, the blends were again weighed and the relative values (%) of the produced CT:COL blends were calculated starting from 60 mg of their precursors (COL 30 mg and CT 30 mg).

### Antimicrobial activity using reference bacteria strains ATCC

In vitro antimicrobial activity was evaluated with the following reference bacteria: *Escherichia coli* ATCC25922, *Staphylococcus aureus* ATCC25923, *Pseudomonas aeruginosa *ATCC27853 and *Listeria monocytogenes* ATCC15313 from the Bacteria Reference Laboratory Oswaldo Cruz Institute (FIOCRUZ, Rio de Janeiro).

For the preparation of the inoculum and evaluation of antimicrobial activity, the reference bacteria were subcultured in Petri dishes containing Mueller–Hinton agar medium (DIFCO^®^) and then incubated at 37 °C for 24 h under aerobic conditions. After the incubation period, isolated colonies were collected and transferred to sterile saline tubes (0.85% NaCl, (w/v)) to obtain the turbidity equivalent to McFarland's Probac 0.5 tube, which corresponds to approximately 1.5 × 108 CFU/mL according to the standardization of the Clinical Laboratory Standard Institute (CLSI) (Clinical and M100 2017).

Antimicrobial activity assays were performed by agar diffusion and broth microdilution tests with an initial concentration of 600 µg/mL of biomaterial. Initially, the blends were evaluated by the well-drilling agar diffusion method according to the recommendation of the CLSI of 2017 (Clinical and M100 2017). The minimum inhibitory concentration (MIC) was determined by the broth microdilution technique using sterile flat-bottom 96-well microplates with a lid. MIC values (in µg/mL) were defined as the lowest concentration capable of completely inhibiting bacterial growth. Ciprofloxacin was used was a positive control and a buffer solution of acetic acid and sodium acetate (pH = 5.53) was used as a negative control. Control experiments of the culture medium and bacterial growth were also performed. The plates were incubated at 35 °C for 24 h under aerobic conditions. Subsequently, the minimum bactericidal concentration (MBC) was obtained. The MBC is defined as the lowest concentration in which the tested blend promoted the death of 99.9% of bacterial cells with respect to the initial inoculum on the surface of the culture medium, i.e., without visible growth on the agar. The experiments were performed in triplicate (Sionkowska et al. 2004).

In vitro cytotoxicity assessment in mouse fibroblasts using the NIH-3T3 cell line from the American Type Culture Collection (ATCC CRL-1658) was performed to verify the biocompatibility of the blends. Cells were maintained in 15 cm^2^ bottles containing DMEM (Dulbecco's Modified Eagle Medium) culture medium supplemented with 10% fetal bovine serum (FBS) and 0.1 mg/mL gentamicin at 37 °C in a humidified 5% atmosphere of CO2. For this test, the samples were previously solubilized in 1 mL of acetic acid and sodium acetate buffer solution and then filtered with 0.22 µm filters to remove particles and maintain sterility. All cytotoxicity tests were performed in triplicate within 48 h using 96-well plates containing 5 × 105 cells per well. Blends were tested at the following concentrations: 15,000, 7,500, 3,750, 750, 75 and 7.5 µg/mL. After the incubation period, the cells were compared with the control and evaluated in terms of their morphology and metabolic activity by the MTT [3- (4,5-dimethylazole-2yl) -2,5-diphenyltetrazolium] and Trypan Blue test.

### Statistical analysis

Statistical analyses were performed for all groups of related experiments and compared to the control group. We used ANOVA followed by Tukey's HSD test with a significance level of α = 0.05.

## Results

**Fig. 1:**
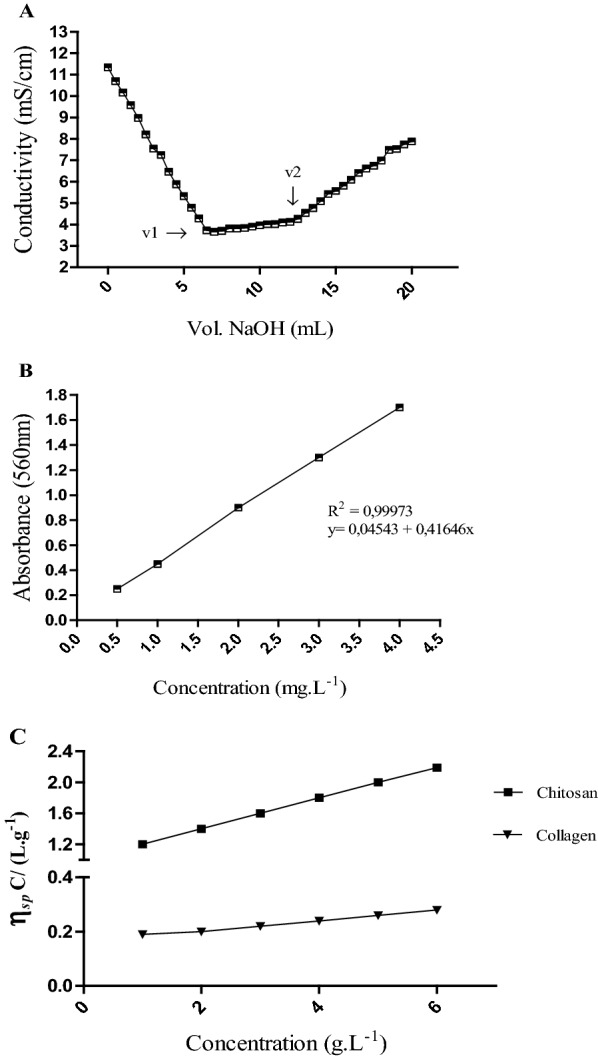
Conductivity titration curve of the chitosan sample (**A**), analytical curve for the determination of 4-hydroxyproline (λ = 560) (**B**), and reduced viscosity curves as a function of the concentration of the biopolymer solutions (**C**)

To characterize the chitosan:collagen (CT:COL) blending process, the degree of chitosan deacetylation was obtained from the conductivity titration curve shown in Fig. 1A. An average degree of chitosan deacetylation of 82%, which characterizes the material as chitosan according to the manufacturer's specifications (75 to 85%), was observed in the sample precipitated with NaOH and excess HCl.

The analytical curves shown in Fig. 1B were obtained via the different 4-Hy concentrations. A correlation coefficient (R2) of 0.999973 was observed when comparing the absorbances for the different concentrations, indicating excellent linearity of the curve. The amount of collagen extracted from the gurijuba swim bladder was 47.56 mg. g-1 and 394.75 mg. g-1 of hydroxyproline and collagen content, respectively. The high concentration of biopolymer indicates that less structural damage occurred during the extraction process.

The relationship between intrinsic viscosity and polymer molar mass was established through the constants k and a based on the Staudinger-Mark-Houwink parameters for aqueoussoluble polymers and biopolymers. From this equation and the intrinsic viscosity values, the chitosan and collagen molar masses were estimated. This variable was important for determining the antimicrobial potential of chitosan employed in this study. The results of viscosity tests for chitosan and collagen obtained from the gurijuba swim bladder are shown in Table 1. For chitosan, considering the degree of deacetylation, solvent and temperature, as well as K = 7.4 × 10^–5^ L.g^−1^ and a = 0.76, the average molar mass was calculated to be 3.7 × 10^5^ g.mol^−1^. For the collagen sample, the parameter values were K = 1.66 × 10^–8^ L.g^−1^ and a = 0.885 and the calculated molar mass was 9.0 × 10^7^ g.mol^−1^. The graph for determining the molar mass of the polymers can be seen in Fig. 1C.

**Table 1:** Viscosity measurements of chitosan and collagen extracted from the Gurijuba swim bladder

Biopolymer	Concentration (g.L^−1^)	Time (s)	Constant (mm^2^/s)	*η*_*r*_ (L.g^−1^)	*η*_*sp*_ (L.g^−1^)	*η*_*red*_ (L.g^−1^)
Chitosan	2	825	0.004	3.41	2.40	1.20
	3	700	0.008	5.78	4.78	1.59
	4	992	0.008	8.19	7.19	1.80
	5	315	0.035	11.38	10.38	2.07
	6	408	0.035	14.75	13.75	2.29
Collagen	2	340	0.004	1.4049	0.4049	0.2024
	3	400	0.004	1.6528	0.6528	0.2176
	4	460	0.004	1.9008	0.9008	0.2252
	5	530	0.004	2.1900	1.1900	0.2380
	6	600	0.004	2.4793	1.4793	0.2465

ηr, Relative viscosity; ηsp, specific viscosity; ηred, reduced viscosity

Considering the probability of adsorbed water in the process of obtaining the products, which could raise doubts about the presence of collagen in the blends, the purchased materials were analyzed with respect to their 4-Hy and collagen content. The results are shown in Table 2. It was observed that collagen was present in all precipitates and that, when extracted from Gurijuba swim bladder, the NaOH precipitation route led to the highest concentrations of hydroxyproline and collagen in the produced biomaterial with values of 6.02 mg.g^−1^ and 48.16 mg.g^−1^, respectively, observed with a CT:COL ratio of 20:80.

**Table 2:** Gurijuba swim bladder hydroxyproline and collagen content of CT: COL blends obtained with the three proportions (20:80, 50:50 and 80:20) via the three preparation routes

Proportion CT:COL	NaOH		NaCl		NaOH/NaCl	
	4-Hy (mg. G^−1^)	COL (mg. G^−1^)	4-Hy (mg. G^−1^)	COL (mg. G^−1^)	4-Hy (mg. G^−1^)	COL (mg. G^−1^)
20:80	6.02^#*^	48.16	4.99	39.92	*3.63*	29.04
50:50	3.83	30.4	0.15	1.2	2.7	21.6
80:20	3.62	28.96	2.95	23.68	3.57	28.56

*CT* chitosan, *COL* collagen, *NaOH* precipitating solution of sodium hydroxide, *NaCl* precipitating solution of sodium chloride, *NaOH/NaCl* precipitating solution combined with sodium hydroxide and sodium chloride

^#^highest value obtained between staging routes

*highest value obtained between chitosan and collagen proportions

**Fig. 2:**
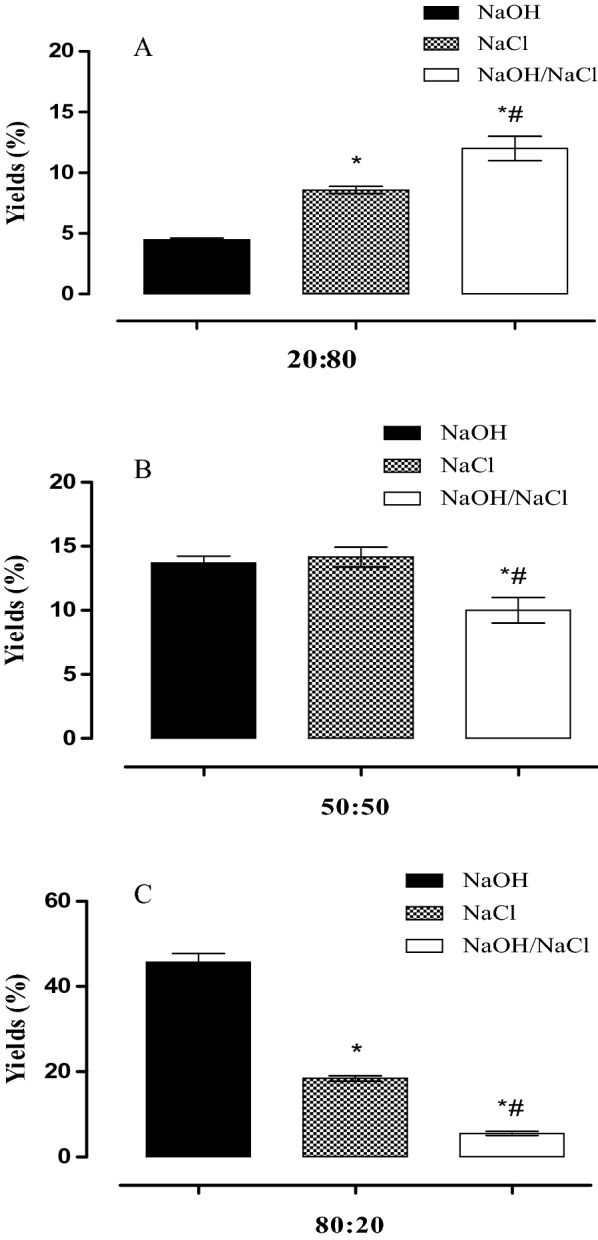
Yield of blends produced from precipitation. Polymer blend CT:COL (20:80) (**A**); Polymer blend CT:COL (50:50) (**B**); Polymer blend CT:COL (80:20) (**C**); NaOH: blend produced in sodium hydroxide precipitating solution; NaCl: mixture produced in sodium chloride precipitating solution; NaOH/NaCl: mixture produced in precipitating solution combined with sodium hydroxide and sodium chloride using ANOVA followed by Tukey’s HSD test

In Table 3 and Fig. 2, the produced biomaterials with the highest yields are shown. The blends produced by the alkaline route with a CT:COL ratio of 80:20 resulted in a 44% yield, which was the highest yield obtained among the tested precipitation methods.

**Table 3:** Yield in % of blends obtained CT: COL from Gurijuba swim bladder for the three proportions (20:80, 50:50, and 80:20) via the three preparation routes

Proportion CT:COL	NaOH	NaCl	NaOH/NaCl
20:80	4.3	8.3	12
50:50	13.3	14	10
80:20	44^#*^	18.2	5.5

*CT* chitosan, *COL* collagen, *NaOH* precipitating solution of sodium hydroxide,  *NaCl* precipitating solution of sodium chloride, *NaOH/NaCl* precipitating solution combined with sodium hydroxide and sodium chloride; (#) highest value obtained between staging routes; (*) highest value obtained between chitosan and collagen proportions

**Fig. 3:**
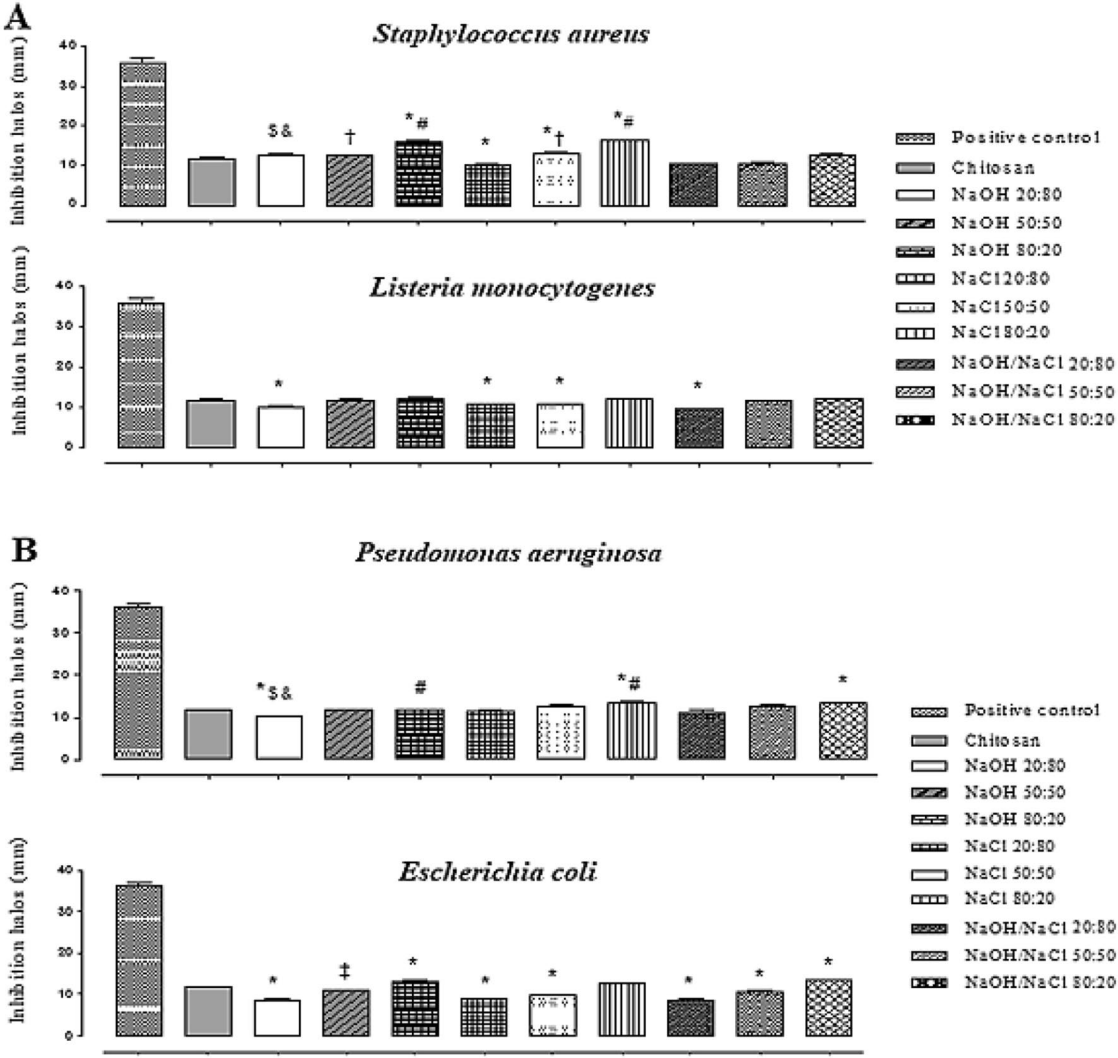
Sensitivity test on Gram-positive (**A**) and Gram-negative (**B**) bacteria. Positive control = ciprofloxacin; NaOH: blend produced in sodium hydroxide precipitating solution; NaCl: mixture produced in sodium chloride precipitating solution; NaOH/NaCl: mixture produced in precipitating solution combined with sodium hydroxide and sodium chloride using ANOVA followed by Tukey’s HSD test

In the microbiological evaluation of CT:COL blends, both gram-negative (*P. aeruginosa* and *E. coli*) and gram-positive (*S. aureus* and *L. monocytogenes*) strains were sensitive to the antimicrobial action of the polymeric blends (p> 0.05), as shown in Tables 4, 5 and 6. Figure 3A and B show the results obtained in the sensitivity test on gram-positive and gram-negative bacteria, respectively. Microbiological analysis showed that the lowest MIC and MBC values were obtained for *S. aureus* (MIC= 4.65 µg.mL^-1^ and MBC= 9.30 µg.mL^-1^) and *P. aeruginosa* (MIC= 9.30 µg.mL^-1^ and MBC= 9.30 µg.mL^-1^). The same strains showed significant results for all tested precipitation methods and proportions. This antibacterial activity was due to the higher amount of chitosan in the biomaterial.

**Table 4:** Antimicrobial evaluation of CT:COL blends at a ratio of 20:80

	Bacteria	NaOH			NaCl			NaOH/NaCl		
		Inhibition zone (mm)	MIC	MBC	Inhibition zone (mm)	MIC	MBC	Inhibition zone (mm)	MIC	MBC
gram-positive	*Staphylococcus aureus* ATCC25923	12.50 ± 0.80	9.30	18.70	10.30 ± 0.10	18.70	18.70	10.58 ± 0.07	9.30	18.70
	*Listeria monocytogenes* ATCC 15,313	10.16 ± 0.15	75.00	150.00	10.66 ± 0.11	150.00	150.00	9.70 ± 0.11	150.00	150.00
gram-negative	*Pseudomonas aeruginosa* ATCC27853	09.73 ± 0.15	9.30	18.70	11.43 ± 0.15	18.70	37.50	10.83 ± 0.76	18.70	18.70
	*Escherichia coli* ATCC 25,922	08.56 ± 0.25	150.00	150.00	08.73 ± 0.15	75.00	75.00	08.53 ± 0.25	75.00	75.00

Inhibition zone: values are presented as the mean ± standard deviation of the mean

*CT* chitosan, *COL* collagen, *NaOH* precipitating solution of sodium hydroxide, *NaCl* precipitating solution of sodium chloride, *NaOH/NaCl* precipitating solution combined with sodium hydroxide and sodium chloride; *MIC* minimal inhibitory concentration; *MBC* minimal bactericidal concentration

**Table 5:** Antimicrobial evaluation of CT:COL blends at a 50:50 ratio

	Bacteria	NaOH			NaCl			NaOH/NaCl		
		Inhibition zone (mm)	MIC	MBC	Inhibition zone (mm)	MIC	MBC	Inhibition zone (mm)	MIC	MBC
gram-positive	*Staphylococcus aureus* ATCC25923	12.60 ± 0.10	4.65	9.30	13.35 ± 0.04	4.65	9.30	10.56 ± 0.15	9.30	9.30
	*Listeria monocytogenes* ATCC15313	11.66 ± 0.32	75.00	75.00	10.73 ± 0.11	75.00	75.00	11.53 ± 0.15	75.00	150.00
gram-negative	*Pseudomonas aeruginosa* ATCC27853	12.63 ± 0.55	9.30	9.30	12.73 ± 0.11	9.30	18.70	11.63 ± 0.25	18.70	18.70
	*Escherichia coli* ATCC25922	10.76 ± 0.15	37.50	37.50	09.63 ± 0.30	37.50	37.50	10.63 ± 0.30	37.50	75.00

Inhibition zone: values are presented as the mean ± standard deviation of the mean

*CT* chitosan, *COL* collagen, *NaOH* precipitating solution of sodium hydroxide, *NaCl* precipitating solution of sodium chloride, *NaOH/NaCl* precipitating solution combined with sodium hydroxide and sodium chloride, *MIC* minimal inhibitory concentration, *MBC* minimal bactericidal concentration

**Table 6:** Antimicrobial evaluation of CT:COL blends at a 80:20 ratio

	Bacteria	NaOH			NaCl			NaOH/NaCl		
		Inhibition zone (mm)	MIC	MBC	Inhibition zone (mm)	MIC	MBC	Inhibition zone (mm)	MIC	MBC
gram-positive	*Staphylococcus aureus* ATCC25923	16.10 ± 0.10	4.65	9.3	16.40 ± 0.10	4.65	9.30	12.30 ± 0.10	9.30	9.30
	*Listeria monocytogenes* ATCC15313	12.26 ± 0.25	75.00	75.00	11.80 ± 0.20	37.5	37.50	12.06 ± 0.11	75.00	75.00
gram-negative	*Pseudomonas aeruginosa* ATCC27853	12.70 ± 0.10	9.30	9.30	13.43 ± 0.32	9.30	18.70	13.23 ± 0.25	9.30	9.30
	*Escherichia coli* ATCC25922	13.00 ± 0.80	37.50	37.50	12.50 ± 0.20	75.00	75.00	13.36 ± 0.20	37.50	37.50

Inhibition zone: values are presented as the mean ± standard deviation of the mean

*CT* chitosan, *COL* collagen, *NaOH* precipitating solution of sodium hydroxid, *NaCl* precipitating solution of sodium chloride, *NaOH/NaCl* precipitating solution combined with sodium hydroxide and sodium chloride, *MIC* minimal inhibitory concentration, *MBC* minimal bactericidal concentration

**Fig. 4:**
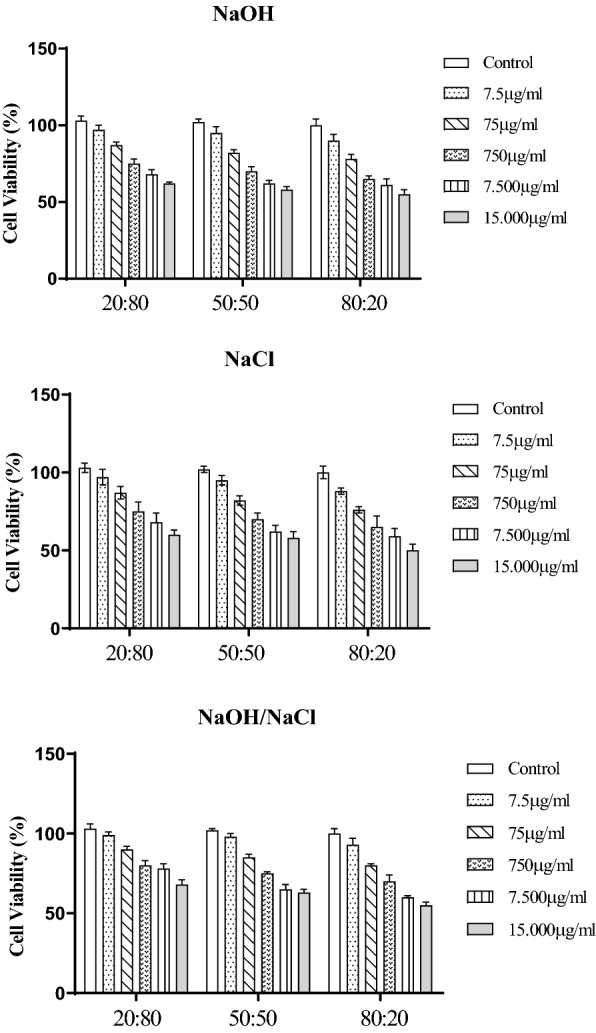
Cytotoxicity assay in mouse fibroblast cells. NaOH: blend produced in sodium hydroxide precipitating solution; NaCl: mixture produced in sodium chloride precipitating solution; NaOH/ NaCl: mixture produced in precipitating solution combined with sodium hydroxide and sodium chloride

The in vitro cytotoxicity evaluation of chitosan:collagen blends was performed to verify the cell viability of the fibroblasts treated with various concentrations of CT:COL blends ranging from 7.5 to 15,000 μg/mL by MTT assay, which was observed to be dose-dependent after 48 h incubation (p > 0.05) (Fig. 4).

## Discussion

The physicochemical results obtained from the characterization of the CT:COL blends suggest that the different precipitation processes of the chitosan and collagen blends directly reflect the efficiency of the production of these biomaterials. Interestingly, the NaOH solution preparation route produced the highest concentrations of hydroxyproline and collagen, although this was expected in the NaCl solution precipitation route since the presence of concentrated saline can favor collagen precipitation (Lima et al. 2006). A study by Chen et al. (Chen et al. 2008) observed that the production process of chitosan and collagen blends influenced the morphology and biological characteristics of each polymer. However, our results showed that the NaOH precipitation route maintained the morphological integrity as well as the biological characteristics of the biopolymers, favoring their molecular interaction.

Our results showed that the most efficient route of preparation employed precipitation in alkaline solution at a ratio of 80:20, which produced the highest yield of biomaterial. However, in highly-concentrated chitosan solutions, the alkaline preparation route is favored due to the molecular interactions between the biopolymers and the coprecipitating collagen during the blend production process. Similar results were obtained in alkaline solutions, where chitosan precipitation was observed by neutralization of protonated amino groups (Fan et al. 2016; Fernandes et al. 2008).

Antimicrobial evaluation of the chitosan/collagen blends in both gram-negative (*P.aeruginosa* and *E. coli*) and gram-positive (*S. aureus* and *L. monocytogenes*) strains revealed that all tested bacteria were sensitive to the antimicrobial action of the polymeric blends observed in the antibiogram, MIC, and MBC. This activity was dependent on the proportion of chitosan in the blend since collagen does not have antimicrobial activity, as observed in the antibiogram. Studies have shown that this activity was directly related to the physicochemical properties of the polymer (such as the %DD, pH and molecular mass) and the membrane characteristics of the microorganisms with negatively charged cell surfaces in grampositive bacteria and more complex cell walls in gram-negative bacteria (Cervera et al. 2004; Vieira et al. 2015).

The %DD of chitosan used in this study indicates the number of polymer’s amino groups arising from the conversion of chitin to chitosan. The higher the ratio of the number of deacetylated units is, the higher is the %DD of the polymer, which directly affects its physicochemical properties and the antimicrobial potential of the material (Möller et al. 2004). Thus, the higher the %DD is, the higher is the concentration of amino groups in chitosan, which, once in contact with physiological fluids, are likely protonated and bind to the anionic groups present in the membranes of these microorganisms, resulting in the agglutination of the cell microbial with chitosan molecules, thus inhibition the bacterial growth. The bacterial sensitivity tests with significant bacterial inhibition of both gram-positive and gram-negative bacteria provide further evidence for this explanation (Möller et al. 2004).

Molecular weight plays na important role in the antimicrobial activity of chitosan. A previous study evaluated the action of chitosan and chitosan oligomers against microorganisms isolated from tofu. The authors found that a chitosan mass of 7.46 × 10^5^ g.mol^−1^ was effective against *E. coli*, 2.24 × 10^5^ g.mol^−1^ was effective against *Pseudomonas fluorescens* and 16.71 × 10^5^ g.mol^−1^ was effective against *L. monocytogenes* and *S. aureus*. In this study, the chitosan molecular mass was 3.7 × 10^5^ g.mol^−1^, demonstrating that we obtained significant bacterial inhibition with a lower molecular weight of chitosan even in the case of gram-positive bacteria (No et al. 2002). In addition, the antimicrobial activity against gram-negative bacteria increased when the molecular mass of chitosan was decreased (Raafat and Sahl 2009).

The results obtained by Zheng and Zhu (Zheng and Zhu 2003) and Li Feng et al. (Qi et al. 2004) indicated a distinct response of chitosan to both types of bacteria. Thus, in gram-negative bacteria, chitosan penetrates the bacterial membrane due to its low molecular mass, resulting in changes in the bacterial metabolism. In contrast, in gram-positive bacteria with a high chitosan molecular, films are formed around the cell, inhibiting its nutrient absorption. However, we obtained significant values for both gram-negative and gram-positive bacteria. Regarding the mechanisms of action of chitosan against the bacterial membrane, the electron micrographs of *S. aureus* and *E. coli* showed that in the presence of a biopolymer, the membrane of *S. aureus* was weakened or even fragmented, while the cytoplasm of *E. coli* was concentrated and the cell interstitium was enlarged (Raafat et al. 2008).

According to a study by Gomes (Gomes 2013), the high antimicrobial activity of high molecular-weight chitosan can be attributed to the pH of the medium in which it is found, thus lower pH values resulting in higher antimicrobial activity of chitosan. The same author found MBC values of *L. monocytogenes* of 800 µg·mL^−1^ at a pH value of 6.5, which was different from the values at a pH value of 5.53 that was used in this study. In addition, we observed more significant MBC results between 37.5 and 150 µg·mL^−1^ for the same microorganism in NaCl precipitation with a CT:COL ratio of 80:20. These findings are in agreement with the results obtained from Dasagrandhi et al. (Dasagrandhi et al. 2018), who found na MIC of 64.0 µg·mL^−1^ for *L. monocytogenes* using unmodified chitosan and ferulic acid-grafted chitosan (AGC).

Chitosan from crabs was evaluated against *S. aureus* and *E. coli*, and they obtained MIC values of 1200 µg·mL^−1^ and 1300 µg·mL^−1^, respectively. In this study the chitosan used presented DD 65%, so obtaining an inhibition zone of 13 mm for *S. aureus* and 10 mm for *E. coli* in the antibacterial activity test (Islam et al. 2011)*.* However, our MIC values were significantly lower in the ratio 80:20 (4.65 µg·mL^−1^ and 37.50 µg·mL^−1^, respectively), besides presented higher inhibition zones (16.10 ± 0.10 for *S. aureus* and 13.00 ± 0.80 for *E. coli*). The lower values in MIC and higher values in inhibition zones are likely associated with the high solubility of chitosan when associated with collagen, as well as the higher DD% of the chitosan used in our study (82%). In this context, Rodrigues (Rodrigues 2017) evaluated high-and low-acetylation chitosans with gelatin (QAG and QBG, respectively) against the bacteria *S. aureus* and found the lowest MBC value for QBG (31.2 μg. mL^−1^). Upon associating his product with jatobá resin, in this study was obtained a reduced MBC value (15.6 μg. mL^−1^). However, in our study, in the tests with *S. aureus*, the polymeric blends, obtained via different preparation routes and different CT:COL proportions, showed a better result (9.3 μg. mL^−1^). Thus, we can observe that when higher DD% and the higher the positive charge after the amino protonation of chitosan, the stronger its antibacterial activity.

Regarding gram-negative bacteria, Mohamed et al. (Mohamed et al. 2019) evaluated seven different modified chitosan hydrogels, obtaining MIC values from 1.95 to 150.00 μg. mL^-1^ for *E. coli* and 15.63 to 175.00 μg. mL^-1^ for *P. aeruginosa*. These results were similar to those found in our study. Consistent with our results, Liu et al. (Liu et al. 2019) evaluated anionic chitosan with high solubility as well as cationic chitosan and obtained MBCs of 8 mg. mL^-1^ and 16.00 mg.mL^-1^, respectively, for *P. aeruginosa*. In addition, a study by Huang et al. (Huang et al. 2019) showed na MBC of 4.00 mg.mL^-1^ for *E. coli* using a sulfonated chitosan (SCS); our results are better when comparing the blends produced in 50:50 and 80:20 (CT:COL) ratios. Thus, our results obtained with the CT:COL blends decreased, significantly, the metabolic activity of gram-positive and gram-negative bacteria, inhibiting their growth depending on the proportion of chitosan in the blend.

In cytotoxicity tests for the evaluation of CT:COL blends, no adverse effects of the blends on fibroblast cells were observed, even at higher doses. This result demonstrated the high biocompatibility of this biomaterial. This result is consistent with the observations made by Kharazian and Vasafi (Akhavan-Kharazian and Izadi-Vasafi 2019), who did not detect toxic characteristics in human fibroblasts after seven days of treatment with chitosan/calcium peroxide films and nanocrystalline cellulose particles.

Previous studies using the chitosan-collagen association in polymeric blend structures have shown biocompatibility, low toxicity, biodegradability, and intrinsic biological activity properties of the synthesized products (Akhavan-Kharazian and Izadi-Vasafi 2019; Kakkar et al. 2014; Martínez-Camacho et al. 2010). Here, we demonstrated that there were no changes in the biological properties of the products prepared via different precipitation routes, which also showed high antimicrobial activity and no cytotoxicity in fibroblast cells. Thus, our polymer blends are biocompatible for use in biomedical applications such as wound healing dressings, tissue engineering, and scaffolds. These findings are consistent with the results found by Kakkar et al. Who found a high cell viability of NIH 3T3 fibroblasts in scaffolds composed of keratin-chitosan–gelatin.

Overall, the results of this study support that the precipitation process for the production of blends composed of chitosan and fish collagen does not alter the biological properties of the polymers, in addition to being a reproducible process. Precipitation in the 80:20 NaOH solution resulted in the highest yield, although it did not lead to higher concentrations of hydroxyproline and collagen in the obtained mixtures. The results of the sensitivity tests performed in bacterial cultures showed that the lowest values of MIC and MBC were obtained for *S. aureus* and *P. aeruginosa* regardless of the precipitation method and the blend proportion. It was demonstrated that the antibacterial activity was due to the higher amount of chitosan in the biomaterial. The cytotoxicity test showed low or no cytotoxic effects in the studied cell line, demonstrating the biomaterial’s biocompatibility and its potential for use in biomedical applications such as wound healing, implants, and scaffolds.

## Data Availability

The datasets generated during and/or analysed during the current study are available from the corresponding author on reasonable request.
